# Understanding the Gut-Kidney Axis in Antineutrophil Cytoplasmic Antibody-Associated Vasculitis: An Analysis of Gut Microbiota Composition

**DOI:** 10.3389/fphar.2022.783679

**Published:** 2022-01-24

**Authors:** Meilian Yu, Lingzhi Li, Qian Ren, Han Feng, Sibei Tao, Lu Cheng, Liang Ma, Shen-Ju Gou, Ping Fu

**Affiliations:** ^1^ Kidney Research Institute, Division of Nephrology, West China Hospital of Sichuan University, Chengdu, China; ^2^ Department of Biostatistics and Data Science, School of Public Health, University of Texas Health Science Center at Houston, Houston, TX, United States

**Keywords:** gut microbiome, ANCA, vasculitis, kidney disease, gut-kidney axis

## Abstract

Increasing evidence suggested that gut microbiota played critical roles in developing autoimmune diseases. This study investigated the correlation between gut microbiota and antineutrophil cytoplasmic antibody-associated vasculitis (AAV) with kidney injury. We analyzed the fecal samples of 23 AAV patients with kidney injury using a 16s RNA microbial profiling approach. The alpha-diversity indexes were significantly lower in AAV patients with kidney injury than healthy controls (Sobs *P* < 0.001, Shannon *P* < 0.001, Chao *P* < 0.001). The beta-diversity difference demonstrated a significant difference among AAV patients with kidney injury, patients with lupus nephritis (LN), and health controls (ANOSIM, *p* = 0.001). Among these AAV patients, the *Deltaproteobacteria, unclassified_o_Bacteroidales,* Prevotellaceae*,* Desulfovibrionaceae *Paraprevotella*, and Lachnospiraceae*_NK4A136_group* were correlated negatively with serum creatinine, and the proportion of *Deltaproteobacteria, unclassified_o_Bacteroidales,* Desulfovibrionaceae*, Paraprevotella, and* Lachnospiraceae*_NK4A136_group* had a positive correlation with eGFR. In conclusion, the richness and diversity of gut microbiota were reduced in AAV patients with kidney injury, and the alteration of gut microbiota might be related with the severity of kidney injury of AAV patients. Targeted regulation of gut microbiota disorder might be a potential treatment for AAV patients with kidney injury.

## Introduction

Antineutrophil cytoplasmic antibody (ANCA)-associated vasculitis (AAV) is a group of systemic diseases characterized by pauci-immune necrotizing inflammation of the small blood vessels, which includes microscopic polyangiitis (MPA), granulomatosis with polyangiitis (GPA), and eosinophilic granulomatosis with polyangiitis (EGPA). ANCAs, mainly targeting at myeloperoxidase (MPO) and proteinase 3 (PR3), were usually the serological markers of AAV. Usually, AAV involves multiple organs, predominantly the lungs and the kidneys (Nakazawa et al., 2019). The overall annual incidence of AAV was about 20 per million with a poor survival rate and high recurrence rate (Watts and Robson, 2018). Most patients with AAV finally suffered from chronic morbidity, including about 35.5% of patients with dialysis-dependent (Hoffman et al., 1992; Flossmann et al., 2011; Binda et al., 2018). Although the pathogenesis of AAV remained not clear, AAV was currently considered to be caused by multifactorial factors, including genetics, environmental factors, and responses of the innate and adaptive immune systems (Geetha and Jefferson, 2020).

Previous studies showed that gut microbiota could affect immune inflammation not only locally at the mucosal level but also systemically (Abt et al., 2012; Clemente et al., 2012). The involvement of gut microbiota in the pathogenesis of autoimmunity diseases has been explored. When the balance between gut microbiota and immune system is disrupted, the uncontrolled inflammatory conditions or a change of tolerance towards microbiota could initiate or promote autoimmunity (Shamriz et al., 2016). Dysbiosis was implicated in many autoimmune diseases, such as rheumatoid arthritis, systemic lupus erythematosus, and inflammatory bowel disease (IBD) (Clemente et al., 2018). For AAV, previous studies had revealed that GPA patients had a higher abundance of *S. aureus* in the respiratory tract, which was associated with the increased relapse risk (Laudien et al., 2010; Glasner et al., 2017; Lamprecht et al., 2019; Wagner et al., 2019).

However, the change of gut microbial in AAV patients with kidney injury remains largely unknown. Therefore, the present study analyzed the gut microbial composition, taxonomic difference, and microbial function prediction of fecal samples from AAV patients with kidney injury by using the 16S rRNA microbial profiling approach.

## Materials and Methods

### Study Participant

We performed a cross-sectional collection of fecal samples from 23 AAV patients with kidney injury, 15 patients with lupus nephritis (LN), and 27 healthy controls in West China Hospital of Sichuan University. Participants with AAV were eligible if they met the modified American College of Rheumatology classification criteria (Jennette et al., 1994; Seeliger et al., 2017). Patients with LN fulfilled the Systemic Lupus International Collaborating Clinics (SLICC) classification criteria and some proven by renal biopsy (Hochberg, 1997; Weening et al., 2004). Fecal samples from 27 healthy donors were collected as healthy controls. Disease activity was evaluated using the clinical composite Birmingham Vasculitis Activity Score (BVAS) V3.0 for patients with AAV and Systemic Lupus Erythematosus Disease Activity Index (SLE-DAI) for those with LN (Bombardier et al., 1992; Suppiah et al., 2011). All participants with AAV, LN, and healthy controls were excluded if they were younger than 18 years or had another systemic inflammatory disorder, known history of HIV or primary immunodeficiency, lymphoma, or other diseases that mimics AAV. Fresh fecal samples were collected in sterile containers and immediately stored at liquid nitrogen within a maximum of three hours from defecation. They were subsequently transported to the laboratory and stored at −80°C until DNA extraction. Written informed consent was obtained from all the participants in the study. Biomedical Ethics Committee of West China Hospital of Sichuan University approved the study (No. 2016–273).

### DNA Extraction and PCR Amplification

Microbial DNA was extracted from 65 fecal samples using the E.Z.N.A.^®^ soil DNA Kit (Omega Bio-tek, Norcross, GA, U.S.) according to the manufacturer’s protocols. The V3-V4 regions of the bacterial 16S rRNA gene sequences were amplified from the diluted DNA extracts with the primers 338F (5′- ACT​CCT​ACG​GGA​GGC​AGC​AG-3′) and 806R (5′-GGACTACHVGGGTWTCTAA T-3′). Polymerase Chain Reaction (PCR) amplification was performed in a 20 μL mixture containing 4 μL of 5 × FastPfu Buffer, 2 μL of 2.5 mM dNTPs, 0.8 μL of each primer (5 μM), 0.4 μL of FastPfu Polymerase and 10 ng of template DNA. The reactions were hot-started at 95°C for 3 min, followed by 27 cycles of 95°C for 30 s, 55 °C for 30 s, and 72°C for 45 s, with a final extension step at 72°C for 10 min. The resulted PCR products were extracted by a 2% agarose gel and further purified using the AxyPrep DNA Gel Extraction Kit (Axygen Biosciences, Union City, CA, USA) and quantified using QuantiFluor™-ST (Promega, USA) according to the manufacturer’s protocol.

### Illumina Miseq Sequencing of 16S rRNA and Data Processing

Purified amplicons were conducted using Illumina MiSeq platforms (Illumina Inc., San Diego, CA) according to the standard protocols by Majorbio Bio-Pharm Technology Co. Ltd. (Shanghai, China). Before assembly, sequences were firstly filtered to remove low-quality or ambiguous reads, including reads with an average quality score <20 over a 50 bp sliding window, reads lacking exact matching with the primer, and reads containing ambiguous character. Operational taxonomic units (OTUs) were clustered with 97% similarity cut off using UPARSE (version 7.1 http://drive5.com/uparse/) and chimeric sequences were identified and removed using UCHIME. The taxonomy of each 16S rRNA gene sequence was analyzed by the RDP Classifier algorithm (http://rdp.cme.msu.edu/) against the Silva (SSU123) 16S rRNA database using a confidence threshold of 70%.

### Statistical Analysis

To test whether gut microbial species could be differentiated among AAV patients with kidney injury, patients with LN, and healthy controls, a metric multidimensional scaling method based on projection known as principal coordinates analysis (PCoA) and the Adonis test were used. We also compared alpha diversity indices across these three groups, such as Sobs, Chao, Shannon, and Simpson., Linear discriminant analysis Effect Size (LEfSe) analysis was used to identify differentially abundant bacteria among the three groups with a cutoff of 2.0. Microbial phylum and genera concentrations among AAV patients with kidney injury, patients with LN, and healthy controls were compared using a Mann-Whitney test. Using a normalized relative abundance matrix, LEfSe performs the Kruskal-Wallis rank sum test to determine the features with significantly different abundances between assigned taxa and uses LDA to assess the effect size of each feature (Segata et al., 2011). Spearman’s correlation was used to investigate the association between the levels of clinical indicators and fecal microbiota composition in AAV patients with kidney injury and visualized by heatmap in R using the “heatmap” package. The PICRUSt was used to construct the Kyoto Encyclopedia of Genes and Genomes (KEGG) Orthology (KO) and KEGG pathway/module profile, predicting functional profiling of microbial communities using 16S rRNA marker gene sequences.

## Results

### General Characteristics

We continuously enrolled 23 AAV patients with kidney injury, including 15 females and 8 males with a median age of 62 years in this study. Among the 23 AAV patients, two were PR3-ANCA positive, twenty were MPO-ANCA positive, and one was both PR3-ANCA and MPO-ANCA positive. The median estimated glomerular filtration rate (eGFR) and serum creatinine were 12.5 ml/min/1.73 m^2^ and 349 μmol/L, respectively. The clinical characteristics of the AAV patients with kidney injury, the patients with LN, and the healthy controls were summarized in Table 1.

**TABLE 1:** Baseline characteristics of subjects included in the study.

**Group 1: Healthy control, *n* = 27**	
Age, years (median, range)	51, 19–73
Sex (f/m)	14/13
Serum creatinine, µmol/L (median, range)	67, 47–88
eGFR, ml/min/1.73m^2^ (median, range)	99.57, 5.9–138.8
**Group 2: AAV, *n* = 23**	
Age, years (median, range)	62, 19–78
Sex (f/m)	15/8
C-ANCA/P-ANCA positive (n/n)	6/17
PR3-ANCA/MPO-ANCA positive (n/n)	3/21
BVAS (median, range)	14, 6–24
Organ involvement (n of ENT/lung/kidney)	2/10/23
Hemoglobin, g/L (median, range)	94, 61–123
Serum creatinine, µmol/L (median, range)	349, 69–683
eGFR, ml/min/1.73 m^2^ (median, range)	12.53, 3.72–108.98
Hypertension, n (%)	16 (69.6)
Diabetes mellitus, n (%)	2 (8.7)
Surgery of gastrointestinal tract, n (%)	1 (4.4)
Gastritis or enteritis, n (%)	3 (13.0)
Treatment, n (%)	
Steroids	15 (65.2)
Immunosuppressant	2 (8.7)
Antibiotic	10 (43.5)
**Group 3: LN, *n* = 15**	
Age, years (median, range)	41, 19–55
Sex (f/m)	11/4
Antinuclear antibody positive, n (%)	15 (100.0)
Anti-double-stranded DNA antibody positive n (%)	6 (40.0)
Anti-Sm positive, n (%)	5 (33.3)
Rheumatoid factor positive, n (%)	2 (13.3)
SLE-DAI (median, range)	10, 5–17
Organ involvement (n of nerve/skin/kidney)	1/1/15
Renal biopsy class, n (%)	
Class II	1 (6.7)
Class III	2 (13.3)
Class IV	6 (40.0)
Pure Class V	1 (6.7)
Class II + V	1 (6.7)
Class III + V	1 (6.7)
Complement C3 <90 mg/dl, n (%)	14 (93.3)
Complement C4 <10 mg/dl, n (%)	5 (33.3)
Hemoglobin, g/L (median, range)	97, 56–123
Serum creatinine, µmol/L(median, range)	76, 52–429
eGFR, ml/min/1.73 m^2^ (median, range)	85.42, 15.76–110.31
Treatment, n (%)	
Steroids	15 (100.0)
Immunosuppressant	9 (60.0)
Antibiotic	7 (46.7)

AAV, ANCA, associated vasculitis; ANCA, anti-neutrophil cytoplasmic autoantibody; MPO, myeloperoxidase; BVAS, birmingham vasculitis activity index, version V3.0; LN, lupus nephritis; HC, healthy controls; PR3, proteinase 3; eGFR, estimated glomerular filtration rate.

### The Richness and Diversity of Gut Microbiota

**FIGURE 1:**
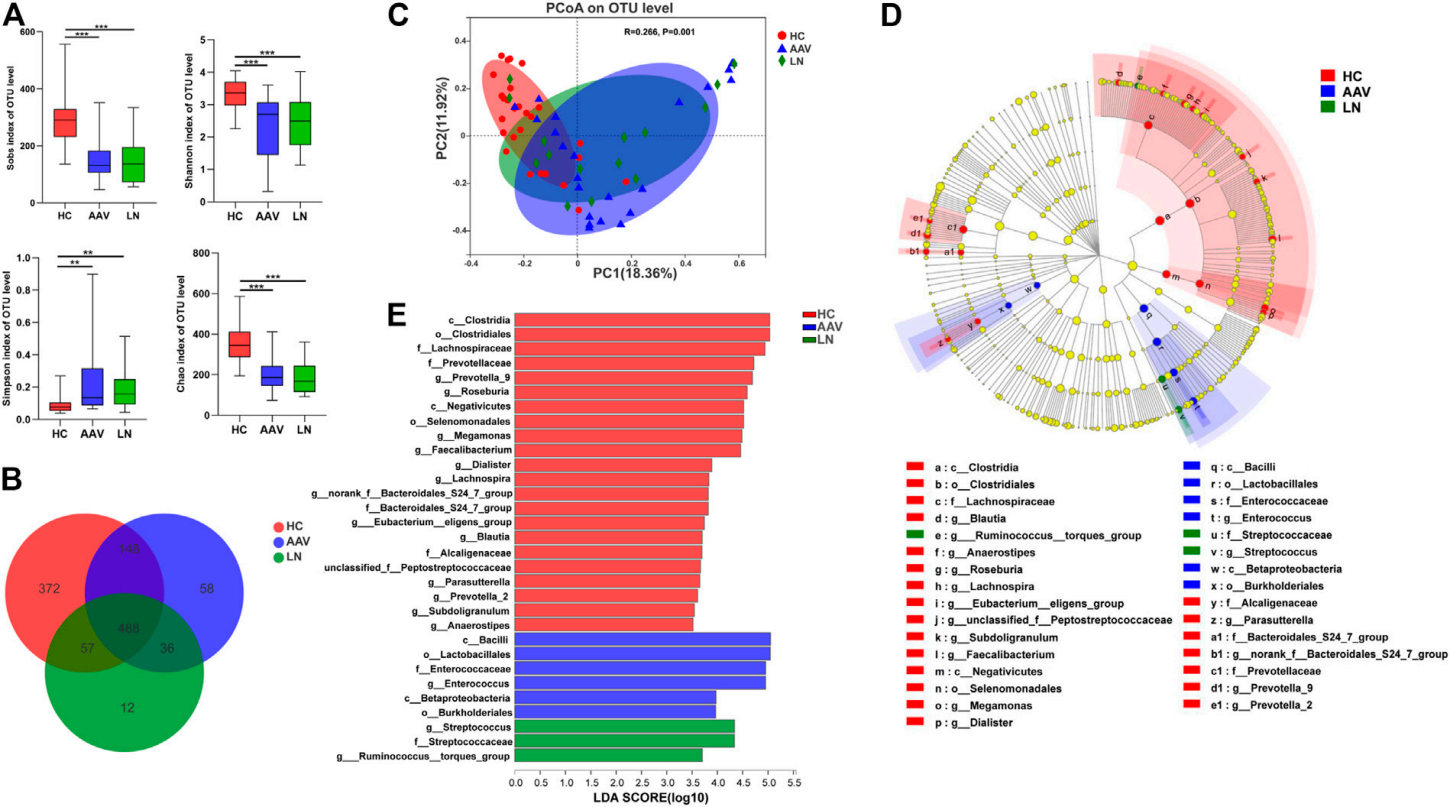
Comparison of gut microbiota of AAV patients, LN patients and healthy controls. **(A)** Alpha diversity indices (Chao, Simpson, Sobs and Shannon) were significantly lower in AAV and LN than that of HC. **(B)** A Venn diagram displaying the overlaps among groups showed that 488 OTUs were shared in all groups, while 58 were unique for AAV. **(C)** The PCoA based on OTUs distribution showed that the gut taxonomic composition was significantly different between AAV and HC. **(D)** The enriched taxa in the AAV, LN and HC gut microbiomes were represented in the cladogram. The central point represents the root of the tree (bacteria) and each ring represents the next lower taxonomic level. **(E)** Crucial bacteria of gut microbiome related to AAV. Based on the LDA selection, 6 genera were significantly enriched compared with LN and HC. *p < 0.05; **p < 0.01; ***p < 0.001. AAV, ANCA associated vasculitis; LN, lupus nephritis; HC, healthy controls; OTUs, operational taxonomic units; PCoA, principal coordinate analysis.

As shown in Figure 1A, the alpha-diversity indexes, including Sobs, Shannon, and Chao indexes, were significantly lower in AAV patients with kidney injury than those of healthy controls (Sobs P < 0.001, Shannon P < 0.001, Chao P < 0.001). At the same time, there were no significant differences in the above indexes between AAV patients with kidney injury and patients with LN. The beta-diversity difference using the Bray Curtis was exhibited in PCoA analysis (Figure 1C), which demonstrated a clear difference among AAV patients with kidney injury, patients with LN, and health controls (ANOSIM, *p* = 0.001).

### Bacterial Community Composition

As exhibited in the Venn diagram (Figure 1B), the number of Operational Taxonomic Units (OTUs) in common between healthy controls and AAV patients with kidney injury was 636, while AAV patients with kidney injury and patients with LN had 524 OTUs in common. The group of AAV patients with kidney injury had 58 specific OTUs, and the group of patients with LN had 12 specific OTUs, which the other two groups did not share. To determine different taxa from phylum to genus level among the three groups, the linear discriminate analysis (LDA) effect size (LEfSe) algorithm was also employed. The LEfSe showed two classes, two orders, one family, one genus were higher in feces of AAV patients with kidney injury and one family, two genera were higher in feces of patients with LN, in comparison with that of healthy controls (Figures 1D,E).

**FIGURE 2:**
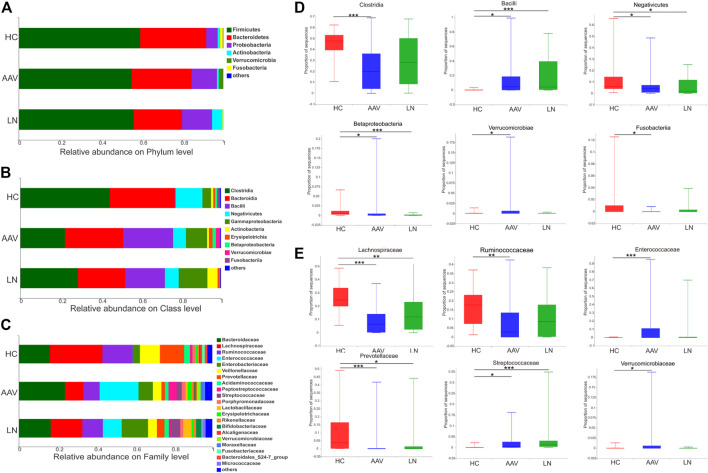
Overview of the bacterial community composition in AAV patients, LN patients and healthy controls. **(A–C)** The relative abundances of sequences classified to major taxonomic phylum, class and family. **(D,E)** Relative abundance of selected taxa among AAV, LN and HC were compared. *p < 0.05; **p < 0.01; ***p < 0.001. AAV, ANCA associated vasculitis; LN, lupus nephritis; HC, healthy controls.

Taxonomic assignment of the OTUs helped to reveal the composition of bacterial population from phylum to genus level separately. At the phylum level, *Firmicutes* was the most predominant bacteria of gut microbiota, followed by *Bacteroidetes* and *Proteobacteria in all groups*. *Firmicutes* and *Bacteroidetes* had a lower proportion in feces of AAV patients with kidney injury and those of LN patients, compared to those of healthy controls. Whereas the abundance of *Proteobacteria* was higher in the feces of AAV patients with kidney injury and LN than that of healthy controls (Figure 2A). At the class level, significant differences were observed in the bacterial composition; the ascendant bacteria in feces of three groups were *Clostridia, Bacteroidia, Bacilli, Negativicutes,* and *Gammaproteobacteria* (Figure 2B). The proportions of *Clostridia, Negativicutes*, *Betaproteobacteria*, and *Fusobacteriia* were lower, while *Bacilli* and *Verrucomicrobiae* were higher in samples of AAV patients with kidney injury compared with those of healthy controls. The decreased species assigned to *Negativicutes* and *Betaproteobacteria* as well as the increased species assigned to *Bacilli* in LN group were compared with healthy controls (Figure 2D). Notably, prominent differences in gut microbiome composition were observed at the family level. The abundance of the bacterial family Enterobacteriaceae, Streptococcaceae*, and* Verrucomicrobiaceae were higher in samples from AAV patients with kidney injury than healthy controls. Conversely, the relative abundance of family Lachnospiraceae, Prevotellaceae*,* and Ruminococcaceae were lower in samples from AAV patients with kidney injury compared with those from healthy controls. (Figures 2C,E). At the genus level, the abundance of *Enterococcus*, *Peptoclostridium, Streptococcus,* and *Akkermansia* was higher in AAV patients with kidney injury compared with healthy controls. However, the proportion of *Faecalibacterium, Prevotella_9,* and *Roseburia,* was lower in samples of AAV patients with kidney injury compared with those of healthy controls (Table 2).

**TABLE 2:** Gut microbiota composition between AAV and HC at the genus level.

Species name	AAV	HC	*P* value
	Median (QR)	Median (IQR)	
g__*Bacteroides*	7722(3–26213)	5553(194–16873)	0.6265
g__*Enterococcus*	63(0–52225)	0(0–261)	<0.0001
g__Faecalibacterium	10(1–10718)	4109(2–11371)	0.0007
g__Prevotella_9	3(0–8191)	1588(1–28002)	<0.0001
g__Escherichia-Shigella	253(0–18027)	248(1–6406)	0.3208
g__Roseburia	2(0–3270)	2844(11–17425)	<0.0001
g__Lachnoclostridium	811(0–8323)	656(145–4690)	0.4595
g__*Streptococcus*	220(0–6763)	28(2–732)	0.0096
g__[Ruminococcus]_gnavus_group	64(0–1989)	83(0–10022)	0.5330
g__Akkermansia	4(0–7146)	0(0–583)	0.0492
g__Dialister	1(0–3440)	4(0–4456)	0.0966
g__Parabacteroides	154(0–3473)	263(2–764)	0.9224
g__*Lactobacillus*	4(0–3732)	5(0–345)	0.9222
g__Parasutterella	0(0–1422)	87(0–2951)	0.0050
g__norank_f__Lachnospiraceae	24(0–758)	325(38–1230)	0.0004
g__Coprococcus_2	0(0–3)	5(0–1053)	<0.0001
g__Lachnospiraceae_NK4A136_group	1(0–1596)	150(0–2387)	0.0010
g__Coprococcus_3	0(0–79)	66(0–398)	<0.0001
g__Paraprevotella	0(0–44)	0(0–9)	0.7499

IQR, interquartile range

### Correlations Between Gut Microbiota and Clinical Characteristics of AAV

**FIGURE 3:**
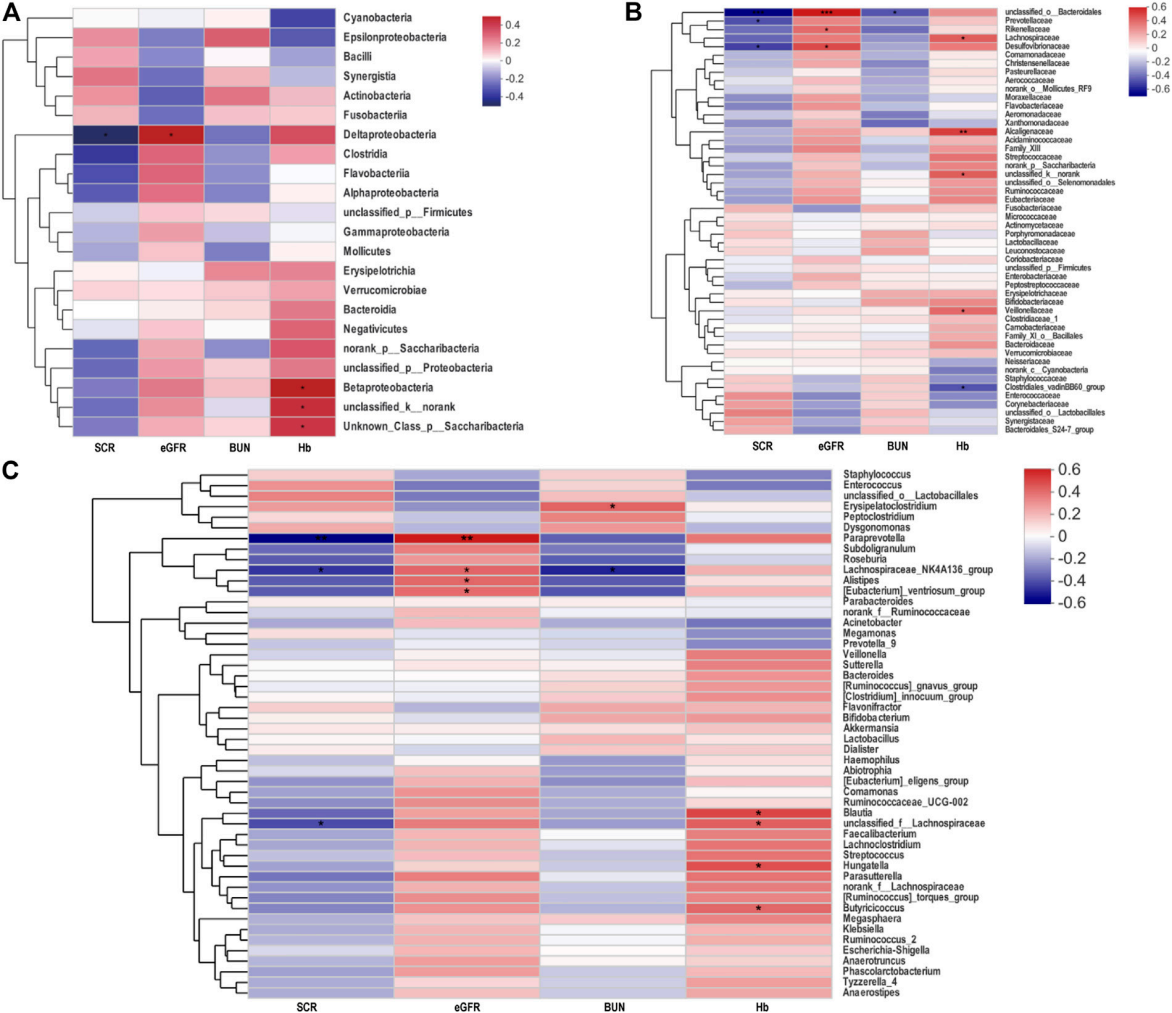
Heatmap of Spearman’s correlation analysis between the gut microbiota of AAV patients and the clinical indices. **(A−C)** Heatmap in class, family and genus levels respectively showed correlations between gut microbiota and biochemical indicators. *p < 0.05; **p < 0.01; ***p < 0.001. AAV, ANCA associated vasculitis; SCR, serum creatinine; eGFR, estimated glomerular filtration rate; BUN, blood urea nitrogen; Hb, hemoglobin.

n order to evaluate whether the proportion of changing gut microbiota was related to serum creatinine (SCR), blood urea nitrogen (BUN), eGFR, and hemoglobin (Hb), the Spearman’s correlation analysis was employed (Figures 3A–C). Among the genera analyzed, at the class, family, and genus level, a negative association between the level of SCR and the proportion of *Deltaproteobacteria, unclassified_o_Bacteroidales,* Prevotellaceae*,* Desulfovibrionaceae *Paraprevotella,* and Lachnospiraceae*_NK4A136_group* was observed. In contrast, the proportion of *Deltaproteobacteria, unclassified_o_Bacteroidales,* Desulfovibrionaceae*, Paraprevotella,* and Lachnospiraceae*_NK4A136_group* had positive correlations with the level of eGFR.

### The Analysis of Gut Microbiota Composition in Samples of Patients With LN and AAV

**FIGURE4:**
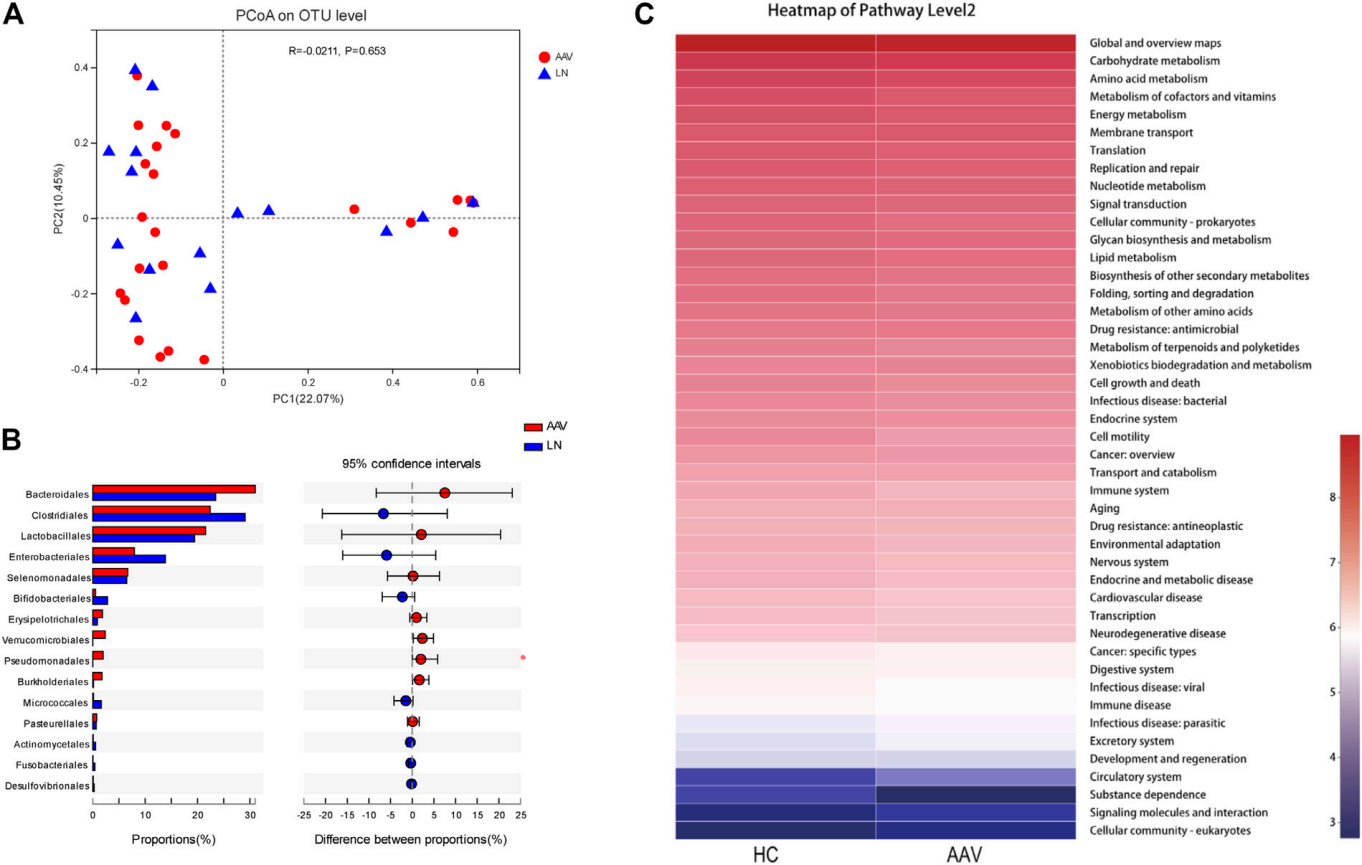
Comparing the microbiota composition between the AAV and LN subjects. **(A)** Beta diversity of gut microbiota showed no obvious significance between AAV and LN. **(B)** The proportion of order *Pseudomonadales* was significantly higher in samples of AAV than that of LN. **(C)** Functional prediction of gut microbiota associated with AAV. KEGG categories were obtained from 16s rRNA gene sequences using PICRUSt. *p < 0.05. AAV, ANCA associated vasculitis; LN, lupus nephritis; HC, healthy controls.

Compared with LN patients, based on Bray-Curtis dissimilarity at OTU level showed that the microbiota composition in samples of AAV patients with kidney injury was similar to that of LN patients (ANOSIM, *p* = 0.653, Figure 4A). In addition, the difference of gut microbiota composition between AAV patients and LN patients was explored using the Mann-Whitney test at different taxon level. There was no apparent difference in the phylum, class, and family levels between samples from AAV patients and LN participants (Supplementary Figures 1A–C). Instead, the proportion of order *Pseudomonadales* was significantly higher in samples of AAV patients with kidney injury patients than that of patients with LN (Figure 4B).

### Metabolic Function of Gut Microbiota

By variance analysis of KEGG metabolic pathways, the differences of metabolic pathways of functional genes in microbiota between the samples of AAN patients with kidney injury and healthy controls could be observed (Figure 4C). Between these two groups in the second-level KEGG pathways, many pathways like “Carbohydrate metabolism”, “Signal transduction”, “Lipid metabolism”, and “Immune disease” were lower in AAV patients with kidney injury than that of healthy controls.

## Discussion

Numerous studies revealed that gut microbiome was closely related to many human diseases for the past several decades (Lynch and Pedersen, 2016). Increasing evidence based on a large body of clinical patients and experimental models indicated that gut microbiome changes also played critical roles in kidney diseases (Hobby et al., 2019; Knauf et al., 2019). Possible explanations had been posted as gut dysbiosis might lead to the gut barrier disruption and thus increase gut permeability, and the influx of endotoxins and uraemic toxins into the kidney *via* the circulation contributed to the renal inflammation. What ‘smore, immune cells originating from the bone marrow encountered dysbiotic microbiota and became overactivated within the intestine. Inflammatory cells, cytokines and soluble urokinase plasminogen activator surface receptor (suPAR) generated in the gut contributed to the renal inflammation *via* the circulation (Yang et al., 2018). However, there were barely studies on gut microbiota in AAV patients with renal injury. whether there was similar mechanism in AAV with renal injury was unknown.

In the present study, we found significant differences in the richness and diversity of gut microbiota in AAV patients with kidney injury compared to the healthy controls. The richness and diversity of gut microbiota were obviously lower in these patients than healthy controls. These results were consistent with the alteration of gut microbiota in inflammatory bowel disease, obesity, and type 2 diabetes mellitus (Le Chatelier et al., 2013; Mar et al., 2016). Typically, the stable gut community formed by gut symbionts resists the invasion of non-native bacteria and the expansion of pathogens, which is called the “colonization resistance” phenomenon. As the diversity of gut bacteria decreases, this phenomenon is disrupted, leading the host to be more susceptible to disease (Ursell et al., 2013; Pickard et al., 2017). However, whether gut dysbacteriosis is involved in the disease occurrence or is the result of the disease needs further investigation.

In the present study, we further explored an increased abundance of *Bacilli* and Enterobacteriaceae in fecal samples of AAV patients with kidney injury than healthy controls. Previous studies showed that *Bacilli* and Enterobacteriaceae were also increased in diseases characterized by immune dysfunction such as Kawasaki disease and inflammatory bowel disease obesity (Darfeuille-Michaud et al., 2004; Kusuda et al., 2014). *Bacilli* could produce a toxin hemolysin BL (HBL), which induced overt inflammation via NLRP3 inflammasome, and Enterobacteriaceae could promote inflammation through stimulating newly recruited monocytes to induce NLRP3-dependent IL-1β release (Seo et al., 2015; Mathur et al., 2019). Interestingly, a previous study in monocytes of AAV patients revealed that NLRP3 induced by FHR1 could secret multiple inflammation factors, such as IL-1β and TNF-α, to trigger inflammatory response of AAV (Irmscher et al., 2019). Therefore, whether *Bacilli* and Enterobacteriaceae are involved in the inflammation of AAV with kidney injury through NLRP3 needs further study.

In the present study, we also investigated that the abundance of *Akkermansia* and Ruminococcaceae was increased in AAV patients with kidney injury. A previous study by Ren et al. (Ren et al., 2020) analyzed and compared the gut microbiome of chronic kidney disease (CKD) patients at different clinical stages and found the two gut microbiotas increased along with the progression of CKD and were correlated with the severity of kidney injury. Another study of kidney injured mouse model by Wang et al. (Wang et al., 2020) found that disordered gut microbiomes could aggravate kidney damage by inducing higher production of uraemic toxins. Therefore, we analyzed the correlation between gut microbiome and clinical indicators of kidney function in AVV. The abundance of Lachnospiraceae*_NK4A136_group* was lower in AAV patients with kidney injury than healthy controls, which was negatively correlated with SCR and positively correlated with eGFR. This suggested that the change of Lachnospiraceae*_NK4A136_group* abundance might be related with the severity of kidney injury. Therefore, modulating gut microbiome disorder, especially Lachnospiraceae*_NK4A136_group changing*, might be a novel intervention to alleviate kidney damage.

In the present study, we further found that the proportion of *Lactobacillus* and *Enterococcus* was higher in AAV patients with kidney injury than healthy controls. More recently, accumulating data indicated that the alteration of gut microbiota also affected the efficacy of cancer immunotherapeutic drugs (Iida et al., 2013; Gori et al., 2019). Cyclophosphamide, as a common immunosuppressor, has an intrinsic “pro-immunogenic” activity to induce the immune response of anti-tumor (Sistigu et al., 2011). A previous study in a tumor mouse model by Viaud et al. (Viaud et al., 2013) found *Lactobacillus* and *Enterococcus* could promote the anti-tumor immune response of cyclophosphamide. For AAV patients with kidney injury, cyclophosphamide also is a first-line therapy drug (Yates et al., 2016). Therefore, whether the alteration of the gut microbiome affects the efficacy of cyclophosphamide in AAV patients with kidney injury is unclear and needs further exploration.

Of note, the study is the first to report the relationship between gut microbiota and AAV patients with kidney injury. However, this study has limitations due to its single-center setting and small sample size. What’s more, since only a few AAV patients did not receive any treatment at the time of fecal sample collection, it was impossible to perform subgroup analysis based on the treatment.

In conclusion, we found reduced richness and diversity of gut microbiota in AAV patients with kidney injury compared with healthy controls. The alteration of gut microbiota might be associated with the severity of kidney injury of AAV patients. Targeted gut microbiota disorder may be the potential as a supplemental option for AAV patients with kidney injury.

## Data Availability

The datasets presented in this study can be found in online repositories. The names of the repository/repositories and accession number(s) can be found below: http://www.ncbi.nlm.nih.gov; PRJNA769047.
