# Supplementary figures and images for: Understanding the Gut-Kidney Axis in Antineutrophil Cytoplasmic Antibody-Associated Vasculitis: An Analysis of Gut Microbiota Composition
Source: Front Pharmacol. 2022 Jan 24;13:783679. doi: 10.3389/fphar.2022.783679 (PMC8819146; doi:10.3389/fphar.2022.783679)

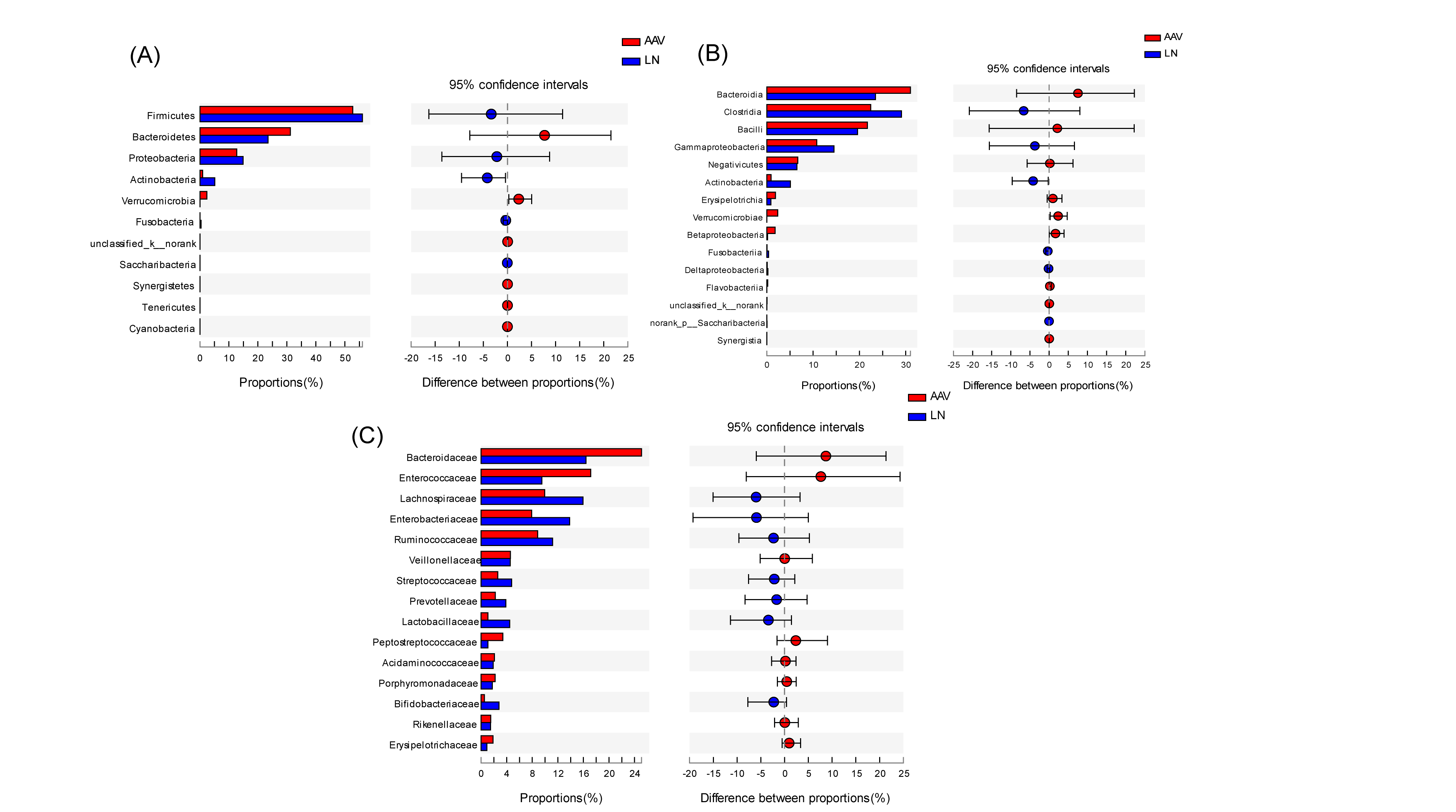

Supplement: Supplementary file 1 [file Image1.TIF]
